# An anion and small molecule inhibition study of the β-carbonic anhydrase from *Staphylococcus aureus*

**DOI:** 10.1080/14756366.2021.1931863

**Published:** 2021-05-31

**Authors:** Linda J. Urbanski, Daniela Vullo, Seppo Parkkila, Claudiu T. Supuran

**Affiliations:** a Faculty of Medicine and Health Technology, Tampere University, Tampere, Finland; b Neurofarba Department, Sezione di Chimica Farmaceutica e Nutraceutica, Università degli Studi di Firenze, Firenze, Italy; c Fimlab Ltd, Tampere University Hospital, Tampere, Finland

**Keywords:** β-carbonic anhydrase, *Staphylococcus aureus*, kinetics, inhibition, anions

## Abstract

Pathogenic bacteria resistant to most antibiotics, including the methicillin-resistant *Staphylococcus aureus* (MRSA) represent a serious medical problem. The search for new antiinfectives, possessing a diverse mechanism of action compared to the clinically used antibiotics, has become an attractive research field. *S. aureus* DNA encodes a β-class carbonic anhydrase, SauBCA. It is a druggable target that can be inhibited by certain aromatic and heterocyclic sulphonamides. Here we investigated inorganic anions and some other small molecules for their inhibition of SauBCA. The halides, nitrite, nitrate, bicarbonate, carbonate, bisulphite, sulphate, stannate, and *N,N*-diethyldithiocarbamate were submillimolar SauBCA inhibitors with *K*_I_s in the range of 0.26 − 0.91 mM. The most effective inhibitors were sulfamide, sulfamate, phenylboronic acid, and phenylarsonic acid with *K*_I_s of 7 − 43 µM. Several interesting inhibitors detected here may be considered lead compounds for the development of even more effective derivatives, which should be investigated for their bacteriostatic effects.

## 1. Introduction

*Staphylococcus aureus* is a Gram-positive bacterium that infects nearly all host tissues in many mammalian species, including humans and livestock, causing severe morbidity and mortality^1^. It belongs to the sadly famous ESKAPE group of bacterial pathogens (*Enterococcus faecium*, *Staphylococcus aureus*, *Klebsiella pneumoniae*, *Acinetobacter baumannii*, *Pseudomonas aeruginosa*, *Enterobacter* spp.) that are resistant to many clinically used antibiotics including methicillin and vancomycin^2^. As a consequence, the treatment of such infection remains particularly challenging if not impossible in severe cases^1^^,^^2^. Thus, there is an urgent need for new classes of antibiotics which can either inhibit the growth of these pathogens and subsequently kill them, or of compounds which can restore the sensitivity of resistant bacteria to the various classes of clinically used agents^3–5^. The inhibitors of the widespread metalloenzyme, carbonic anhydrase (CA, EC 4.2.1.1), were recently shown to be effective in inhibiting the growth (possessing a significant bactericidal activity) of some drug-resistant pathogens, such as vancomycin-resistant *Enterococci*^5^ and *Neisseria gonorrhoeae*^6^.

In fact, CAs are present in most microorganisms including bacteria and are encoded by at least four genetic families (although new ones may still exist to be reported), which are the α-, β-, γ-, and ι-CAs^4^^,^^7^^,^^8^. In some bacteria, such as *Escherichia coli*, the CAs are essential for the survival of the organism^8^. For others, such as *Helicobacter pylori*^4^, the CAs assure the acclimation of the bacterium in the specific niches (gastric and duodenal mucosa) in which it thrives, whereas for others, such as *Vibrio cholerae*, these enzymes participate in the secretion of bicarbonate which is a virulence factor of this pathogen^7^. In the last decade, many representatives of these enzymes, belonging to all four classes present in bacteria, were cloned and characterised both biochemically and structurally in the search for inhibitors. This can eventually lead to the development of new antibacterial agents. Among the various species which have been characterised in this way are *E. coli*, *H. pylori*, *Mycobacterium tuberculosis*, *Vibrio cholerae*, *Pseudomonas aeruginosa*, *Porphyromonas gingivalis*, *Streptococcus* spp., *Staphylococcus aureus*, etc.^4^^,^^7–15^. Although the scientific community was rather sceptical for a long time that bacterial CA inhibition may lead to significant growth inhibition of pathogenic bacteria, Flaherty’s group recently published the long-awaited^5^^,^^6^ proof-of-concept that inhibition of bacterial CAs may lead to antibiotics with novel mechanisms of action. They showed that the sulphonamide CA inhibitor (CAI) acetazolamide and some of its derivatives, as well as dorzolamide, outperformed the current drug of choice, linezolid, both *in vitro* and *in vivo*, for inhibiting the growth of vancomycin-resistant enterococci (VRE)^5^ and *N. gonorrhoeae*^6^. Furthermore, other groups have demonstrated that CAIs may exhibit reduced potential for the development of drug resistance, as in the case of *H. pylori* and ethoxzolamide as CAI. Mutations were observed in several bacterial genes, including the bacterial α-CA gene, but the pathogen remained susceptible to the drug at clinically relevant concentrations^9^.

Recently, we cloned and characterised a β-CA of *S. aureus* (SauBCA), an enzyme that possesses a high catalytic activity for the physiologic CO_2_ hydration reaction to bicarbonate and protons, with the following kinetic parameters: *k*_cat_ of 1.46 × 10^5^ s^−1^ and a *k*_cat_*/K*_M_ of 2.56 × 10^7^ s^–1 ^M^−1^. This enzymatic function was inhibited by various sulphonamide derivatives, which represent one of the main classes of inhibitors of these enzymes^16^. In this study, we continue the exploration of the inhibitors of SauBCA, reporting its inhibition profile with anions and other small molecules known to inhibit CAs.

## 2. Materials and methods

### 2.1. Chemistry

Anions and small molecules were commercially available reagents of the highest available purity from Sigma-Aldrich (Milan, Italy). Purity of tested compounds was higher than 99%.

### 2.2. Enzymology

SauBCA was a recombinant enzyme obtained in-house as described earlier^15^.

### 2.3. CA activity and inhibition measurements

An Applied Photophysics stopped-flow instrument was used for assaying the CA catalysed CO_2_ hydration activity^17^. Phenol red at a concentration of 0.2 mM was used as a pH indicator (working at the absorbance maximum of 557 nm) with 10 mM Hepes (pH 7.4) as a buffer, and in the presence of 10 mM NaClO_4_ for maintaining constant ionic strength. The initial rates of the CA-catalysed CO_2_ hydration reaction were followed for a period of 10 − 100 s. The CO_2_ concentrations ranged from 1.7 to 17 mM for the determination of the kinetic parameters and inhibition constants. For each inhibitor, at least six traces of the initial 5 − 10% of the reaction were used for determining the initial velocity. The uncatalyzed rates were determined in the same manner and subtracted from the total observed rates. Stock solutions of inhibitors (10 − 20 mM) were prepared in distilled-deionized water and dilutions up to 0.01 µM were done thereafter with the assay buffer. Inhibitor and enzyme solutions were preincubated together for 15 min at room temperature prior to assay in order to allow for the formation of the enzyme-inhibitor complex. The inhibition constants were obtained by non-linear least-squares methods using PRISM 3 and the Cheng–Prusoff equation, whereas the kinetic parameters for the uninhibited enzymes were obtained from Lineweaver-Burk plots, as reported earlier^18–20^. The results represent the mean from at least three different determinations (data not shown). The SauBCA concentration in the assay system was 9.7 nM.

## 3. Results and discussion

Inorganic anions represent a well-characterised class of CAIs^21^. Our study included anions known to have a high affinity in solution for complexing metals, such as halides and especially pseudohalides (cyanide, cyanate, thiocyanate, azide, etc.), as well as those which do not easily form complexes with transition metal ions (e.g. sulphate, selenate, tellurate, tetraborate, etc.). Both groups of anions have been shown to possess inhibitory action against all classes of CAs investigated so far, from prokaryotes to eukaryotes^21–23^. Furthermore, small molecules such as sulfamide, sulphamic acid, phenylboronic, and phenylarsonic acid also possess such properties^24^. In this study, we investigated a panel of such anions and small molecules for the inhibition of SauBCA (Table 1). The inhibition data of the abundant human (h) isoforms hCA I and II as well as those of another bacterial CA, NgCA from *N. gonorrhoeae*^25^ are also shown in Table 1 for comparison.

**Table 1.** Inhibition constants (*K*_I_s) of anion inhibitors against hCA I, II and the bacterial enzymes NgCA and SauBCA, measured by a stopped-flow CO_2_ hydration assay^17^.

Anion^b^	*K*_I_ (mM)^a^			
	hCA I	hCA II	NgCA	SauBCA
F^–^	>300	>300	8.3	0.48
Cl^–^	6	200	4.8	0.69
Br^–^	4	63	4.0	0.26
I^–^	0.3	26	9.6	0.72
CNO^–^	0.0007	0.03	0.43	3.7
SCN^–^	0.2	1.6	0.92	28.6
CN^–^	0.0005	0.02	1.0	4.1
N_3_^–^	0.0012	1.51	2.1	7.4
NO_2_^–^	8.4	63	0.59	0.56
NO_3_^–^	7	35	0.85	0.41
HCO_3_^–^	12	85	1.3	0.42
CO_3_^2–^	15	73	2.9	0.76
HSO_3_^–^	18	89	0.66	0.90
SO_4_^2–^	63	>200	0.83	0.91
HS^–^	0.0006	0.04	0.55	19.3
NH_2_SO_2_NH_2_	0.31	1.13	0.058	0.009
NH_2_SO_3_H	0.021	0.39	0.024	0.043
PhAsO_3_H_2_	31.7	49	0.74	0.007
PhB(OH)_2_	58.6	23	0.15	0.008
ClO_4_^–^	>200	>200	>100	>100
SnO_3_^2–^	0.57	0.83	1.7	0.32
SeO_4_^2–^	118	112	0.87	4.8
TeO_4_^2–^	0.66	0.92	0.76	42.0
OsO_5_^2–^	0.92	0.95	2.3	6.0
P_2_O_7_^2–^	25.8	48	4.9	>100
V_2_O_7_^2–^	0.54	0.57	2.8	>100
B_4_O_7_^2–^	0.64	0.95	0.65	8.7
ReO_4_^–^	0.11	0.75	0.96	>100
RuO_4_^–^	0.101	0.69	1.9	>100
S_2_O_8_^2–^	0.107	0.084	0.79	>100
SeCN^–^	0.085	0.086	0.66	5.5
NH(SO_3_)_2_^2–^	0.31	0.76	0.25	>100
FSO_3_^–^	0.79	0.46	0.61	8.9
CS_3_^2–^	0.0087	0.0088	0.088	11.4
EtNCS_2_^–^	0.00079	0.0031	0.0051	0.64
PF_6_^–^	>100	>100	>100	>100
CF_3_SO_3_^–^	>100	>100	5.7	>100

^a^Mean from three different assays, measured by a stopped-flow technique (errors were in the range of ± 5–10% of the reported values); ^b^As sodium salts, except sulphamide and phenylboronic acid.

The following observations can be delineated from the data presented in Table 1 regarding the inhibition of SauBCA with anions and small molecules:

- anions with a rather low propensity for complexating metal ions, such as perchlorate and hexafluorophosphate, and triflate, did not inhibit SauBCA significantly with concentrations up to 100 mM in the assay system. This is also the case for their interaction with hCA I and II, as well as many other CAs belonging to all known classes. For this reason, we used perchlorate at 10 mM concentration for maintaining constant ionic strength in the stopped-flow assays, as mentioned in Materials and methods. Other anions, such as pyrodiphosphate, divanadate, perruthenate, perrhenate, peroxydisulfate and iminidisulfonate, were also in this category of non-inhibiting anions. It should be noted, however, that some of them act as rather efficient anion inhibitors of other enzymes than SauBCA, as shown in Table 1.
- The following anions showed weak inhibitory action against SauBCA: thiocyanate, hydrogensulfide, tellurate, and trithiocarbonate, with inhibition constants in the range of 11.4–42 mM (Table 1). Except for tellurate, which is not a high-affinity ligand for metal ions, the other three anions mentioned here are either very good coordinating agents for transition metal ions (thiocyanate, hydrogensulfide, and trithiocarbonate) or quite effective CAIs (see the trithiocarbonate data for hCA I, II and NgCA in Table 1). Additionally, in some cases, the X-ray crystal structure of their complexes with hCA II is also available^26^^,^^27^. Thus, these low inhibition constants against SauBCA deserve a better investigation in order to understand the structural features of this enzyme active site, which for the moment has not been crystallised.
- Effective, millimolar inhibition was observed for the following anions: cyanate, cyanide, azide, selenate, perosmate, tetraborate, selenocyanate, and fluorosulfonate, with *K*_I_s in the range of 3.7–8.9 mM. It should be noted that some of these anions (e.g. cyanide, cyanate) are extremely potent, micromolar hCA I inhibitors, whereas their activity against hCA II and NgCA are usually in the millimolar or submillimolar range.
- The halides, nitrite, nitrate, bicarbonate, carbonate, bisulphite, sulphate, stannate, and *N,N*-diethyldithiocarbamate were even more effective as SauBCA inhibitors with K_I_s in the range of 0.26–0.91 mM (Table 1). Among the halides, bromide was the most effective inhibitor, whereas the isosteric/isoelectronic nitrate and bicarbonate had very similar inhibitory behaviour. Sulphate, which is an extremely weak hCA I and II inhibitor, is on the other hand much more effective as an inhibitor of bacterial CAs. In fact, many such bacterial enzymes have been purified in the presence of extremely high concentrations of sulphate and showed no catalytic activity due to inhibition by the anion present in the buffer or the assay system^14^.
- The most effective inhibitors detected in the current study were sulfamide, sulfamate, phenylboronic acid, and phenylarsonic acid, which showed *K*_I_s in the range of 7–43 µM. In fact, these compounds are known to inhibit many CAs of different classes, and X-ray crystal structures have even been reported for some of the enzyme-inhibitor complexes^12^^,^^28^.

## 4. Conclusions

SauBCA is a high activity β-CA present in the genome of the bacterial pathogen *S. aureus*, known for its extensive drug resistance to classical antibiotics. We investigated here its inhibition with a series of inorganic and organic anions. Perchlorate, hexafluorophosphate, triflate, pyrodiphosphate, divanadate, perruthenate, perrhenate, peroxydisulfate, and iminidisulfonate did not show any significant inhibitory action against this enzyme with concentrations up to 100 mM in the assay system. Thiocyanate, hydrogensulfide, tellurate, and trithiocarbonate were weak inhibitors with *K*_I_s in the range of 11.4 − 42 mM, whereas cyanate, cyanide, azide, selenate, perosmate, tetraborate, selenocyanate, and fluorosulfonate showed *K*_I_s in the range of 3.7 − 8.9 mM. The halides, nitrite, nitrate, bicarbonate, carbonate, bisulphite, sulphate, stannate, and *N,N*-diethyldithiocarbamate were more effective as SauBCA inhibitors with *K*_I_s in the range of 0.26 − 0.91 mM, but the most effective inhibitors were sulfamide, sulfamate, phenylboronic acid, and phenylarsonic acid, which showed *K*_I_s in the range of 7 − 43 µM. Several inhibitors detected here may be considered as lead compounds for the development of even more effective derivatives, which should thereafter be investigated for their bacteriostatic effects.
